# Efficacy of a Once-Daily Supplement in Managing Canine Chronic Kidney Disease

**DOI:** 10.3390/ani15192884

**Published:** 2025-10-02

**Authors:** Francesca Perondi, Alessio Ruggiero, Monica Isabella Cutrignelli, Ilaria Lippi, Giorgia Meineri, Elisa Martello

**Affiliations:** 1 Department of Veterinary Science, University of Pisa, San Piero a Grado, 56122 Pisa, Italy; f.perondi87@gmail.com (F.P.); ilaria.lippi@unipi.it (I.L.); 2 Department of Veterinary Medicine and Animal Production, University of Naples Federico II, 80137 Naples, Italy; alessio.ruggiero@unina.it (A.R.); monica.cutrignelli@unina.it (M.I.C.); 3 Department of Veterinary Sciences, School of Agriculture and Veterinary Medicine, University of Turin, 10095 Torino, Italy; giorgia.meineri@unito.it; 4 Centre for Evidence Based Healthcare, School of Medicine, University of Nottingham, Nottingham NG5 1PB, UK

**Keywords:** CKD, dogs, dietary supplement, hyperphosphatemia, once-daily medication

## Abstract

Background: Chronic kidney disease (CKD) in companion animals leads to progressive renal deterioration and metabolic complications such as hyperphosphatemia and metabolic acidosis, particularly in advanced stages. Methods: This 90-day, double-blind, randomized controlled study evaluated the effects of a renal supplement (Renal Combi, Candioli srl, Beinasco, Turin, Italy) in 30 dogs diagnosed with CKD. Dogs were randomly assigned to a treatment group (TRT, *n* = 15), receiving a commercial renal diet plus the supplement (once daily), or a control group (CTR, *n* = 15), receiving the same diet and a placebo. Results: Results showed that supplementation effectively reduced proteinuria, blood urea, and phosphorus levels, stabilized creatinine and SDMA, and maintained blood pressure. It also improved markers of systemic inflammation and oxidative stress. Conclusions: The once-daily dosage simplified administration and was especially beneficial for hyporexic dogs or those on multiple medications. This study supports the use of the tested supplement as an effective adjunctive therapy for managing CKD in dogs, offering metabolic, renal, and nutritional benefits while enhancing compliance through convenient dosing.

## Simple Summary

Chronic kidney disease (CKD) leads to irreversible loss of kidney function in dogs, often progressing with metabolic complications. This double-blind, randomized controlled study evaluated the efficacy of a once-daily renal supplement (Renal Combi, Candioli srl, Beinasco, Turin, Italy) in managing advanced CKD. Thirty dogs were enrolled and assigned to either a treatment or control group. Hematologic, biochemical, and urinalysis assessments were conducted throughout the study. The supplement demonstrated beneficial effects, including improved control of uremia, phosphate levels, blood pressure, inflammation, and oxidative stress. The simplified once-daily administration improved compliance, making it a practical adjunctive therapy for managing canine CKD.

## 1. Introduction

Chronic kidney disease (CKD) in companion animals is characterized by the progressive loss of functional renal tissue, often leading to significant metabolic disturbances such as hyperphosphatemia and metabolic acidosis—particularly in the later stages of the disease [1,2,3,4,5] (see Figure S1).

Hyperphosphatemia contributes to the progression of CKD and is a key driver of complications such as renal secondary hyperparathyroidism and renal osteodystrophy. It is also directly associated with increased mortality in affected animals [6,7,8,9,10]. Consequently, controlling serum phosphorus levels is a central therapeutic goal in CKD management. Management usually starts with dietary phosphorus restriction, followed by the introduction of phosphate binders if dietary measures alone are inadequate [3,11,12,13].

Nutritional management remains a cornerstone of chronic kidney disease (CKD) treatment in veterinary medicine. However, managing patients with advanced CKD is often complicated by hyporexia or anorexia, which can hinder both dietary and pharmacologic strategies [11,14,15]. Achieving improvements in body condition score (BCS) and overall nutritional status in these cases represents a significant therapeutic success. Administering multiple oral medications under such conditions poses additional challenges, often requiring treatment adjustments to account for decreased patient compliance and tolerance [11].

A recent study reported promising results from a novel dietary supplement (Renal Combi, Candioli srl, Beinasco, Turin, Italy) that addresses several CKD-related complications, including phosphate retention, acid–base imbalances, hypertension, inflammation, and oxidative stress in dogs with advanced renal disease (Martello, 2021) [16]. This study is a double-blind, randomized controlled trial that aims to test the efficacy of the same supplement in dogs with advanced CKD (IRIS stages 3 and 4), with a specific emphasis on reducing to a once-daily administration while preserving therapeutic efficacy. Notably, a once-daily formulation may provide a significant clinical benefit, especially for dogs and cats with advanced CKD, who often have reduced appetite, by enhancing treatment compliance and overall disease management.

## 2. Materials and Methods

### 2.1. Study Design

A double-blind, randomized controlled study was performed in dogs diagnosed with chronic kidney disease (CKD). Diagnosis was based on the presence of persistent azotemia, characteristic clinical signs (including progressive weight loss, poor appetite, and/or polyuria-polydipsia (PU/PD)), and ultrasonographic evidence of chronic structural renal abnormalities. CKD staging was performed in accordance with the guidelines of the International Renal Interest Society (IRIS) [13]. The experimental unit in the study was considered the single dog. The sample size was designed in order to guarantee a power of the study greater than 75% and a confidence of 90%. Sample size was previously calculated with the Altman’s nomogram, considering the serum creatinine (CREA) levels as the primary endpoint. Dogs with concurrent conditions that could confound the diagnosis or progression of CKD were excluded. The patients included in the study presented with conditions such as impaired renal perfusion causing pre-renal azotemia, urinary tract obstruction of post-renal origin, acute kidney injury, genitourinary tract infection or inflammation, neoplastic processes, hypothyroidism, chronic heart failure, and diabetes mellitus. All dogs were fed the same commercial renal diet (Monge Renal), and the diet was introduced 15 days prior to the start of the trial. Dogs with persistent proteinuria or a history of hypertension received standard medical treatment for at least 30 days prior to the start of the trial. All dogs receiving antihypertensive or antiproteinuric therapy were clinically stable at enrollment. Their treatments had been consistent for at least 30 days before inclusion and were maintained unchanged throughout the study. The veterinarian determined treatment based on IRIS staging guidelines, and the dogs continued participation in the trial. The duration of the study was set as 90 days.

### 2.2. Animals

A total of 30 dogs were enrolled in the trial and randomly assigned to either a treatment group (TRT, *n* = 15) or a control group (CTR, *n* = 15). Both investigators and owners were blinded to group allocation until the conclusion of the study. All owners were fully informed of the study procedures and provided written informed consent prior to participation. The experimental protocol was approved (Prot.n. 708, 17 March 2021) by the Ethical Committee of the Department of Veterinary Sciences, University of Turin (Italy).

### 2.3. Veterinary Evaluations

A comprehensive clinical evaluation, along with blood and urine tests, was performed at baseline (T0) and subsequently at days 30 (T30), 60 (T60), and 90 (T90) of the study. The clinical examination included simultaneous measurement of body weight (BW; pet scale, four sensors, maximum 100 kg, d1/4100 g; Momert, Dunaujvaros, Papírgyáriút 12, 2400, Fejer, Hungary) and assessment of body condition score (BCS; 1 to 5 scoring system). All evaluations were conducted by one veterinarian to ensure consistency. Systolic (SBP) and diastolic blood pressure (DBP) were measured (electronic sphygmomanometers-CONTEC, Osaka, Japan), with five consecutive readings taken; the mean of these values was recorded for analysis [17]. A complete blood count (CBC) was performed using an automated veterinary hematology analyzer (scil Vet abc™, Grosseto, Italy) and included hematocrit (HCT), hemoglobin (Hb), red blood cell (RBC), white blood cell (WBC), neutrophil (N), eosinophil (EO), basophil (B), lymphocyte (LYM), and platelet (PLT) levels. The serum biochemistry panel (Automatic Analyzer-Echo, Edif, Roma, Italy) included the evaluation of blood urea nitrogen (BUN), creatinine (CREA), phosphorus (P), total protein (TP), albumin (ALB), albumin/globulin ratio (A/G), and symmetric dimethylarginine (SDMA). Venous blood gas analysis (Abaxis VetScan i-Stat1, Abbott, Chicago, IL, USA) was performed to assess ionized calcium (iCa). Urine samples were collected and analyzed according to the methodology described by Biasibetti et al. [18]. Urine protein (UP) and urinary specific gravity (USG) were measured using the pyrogallol red method and the Jaffè method, respectively [19]. Urine protein-to-creatinine ratio (UPC) was subsequently calculated. In addition, inflammatory and oxidative stress markers were analyzed, including C- reactive protein (CRP, a canine-specific ELISA kit, BD Biosciences, BioTek Instruments, Winooski, VT, USA) and d-ROMs (Free Carpe Diem, Diacron International srl, Grosseto, Italy) levels.

### 2.4. Supplement

To minimize variability due to dietary composition, all dogs were fed the same commercial renal diet (Monge Renal) throughout the study. The diet was introduced fifteen days prior to the start of the trial to allow for acclimatization. Each dog received an individualized feeding plan based on its metabolic needs, calculated according to the FEDIAF Nutritional Guidelines (Fédération Européenne de l’Industrie des Aliments pour Animaux Familiers) [20]. Daily food intake was determined based on the resting energy requirement (RER), calculated using the following formula:

RER (kcal/day) = 70 × (body weight in kg) ^ 0.75

The RER was then multiplied by a life-stage and activity-specific factor to estimate the maintenance energy requirement (MER), as outlined in the FEDIAF guidelines [20].

Dogs assigned to the TRT group received a commercial diet supplemented with Renal Combi (Candioli srl, Beinsaco, Turin, Italy). The supplement is composed of several active ingredients, including chitosan, sodium bicarbonate, calcium carbonate, calcium lactate-gluconate, *Lactobacillus acidophilus* D2/CSL, *Olea europaea* L. extract, and fructooligosaccharides (see Table 1) [16]. Calcium-based compounds such as calcium carbonate and calcium lactate-gluconate help limit intestinal phosphorus absorption by binding it within the gut. Chitosan contributes by decreasing the uptake of phosphorus and nitrogenous waste products, while also offering antioxidant benefits through its ability to neutralize free radicals. Sodium bicarbonate serves as a systemic alkalinizing agent, aiding in the correction of metabolic acidosis by increasing serum bicarbonate levels, pH, and partial CO_2_ pressure. *L. acidophilus* and fructooligosaccharides enhance gastrointestinal health by modulating the microbiota, which in turn may reduce nitrogenous waste production. Additionally, the extract of *Olea europaea* L. is known for its antioxidative and antihypertensive properties. The CTR group received the same commercial diet with a placebo (Table 1). Both the powdered supplement and the placebo were administered once daily with food at a dosage of 0.2 g/kg body weight. Owners were instructed to monitor and record any adverse events, such as vomiting, diarrhea, or loss of appetite, throughout the trial.

### 2.5. Statistical Analysis

The statistical analysis was conducted using R software ver. 4.4.1 [21]. The impact of the supplement on the parameters documented during the study was analyzed using a variance analysis according to a repeated measures model:

y = µ + G_j_ + T_k_ + GT_jk_ + S_i_ + ε_jki_

where y is the dependent variable, µ is the overall mean, G_j_ is the fixed effect of the jth diet, T_k_ is the fixed effect of the kth time, GT_jk_ is the fixed effect of the interaction between the jth diet and the kth time, S_i_ is the random effect of the ith subject, and ε_jki_ is the error. Furthermore, a post hoc test (Tukey’s test—HSD) was utilized to conduct both intra-group and between-group comparisons at the experimental times. The analysis of ionized calcium (iCa) was conducted utilizing Wilcoxon’s non-parametric test. The significance level used for multiple comparisons for the repeated measures was set as *p* < 0.05.

## 3. Results

The TRT group showed a significantly (*p* < 0.0001) higher BW at T2 and T3 compared to T0, while no significant differences during the trial were observed in the CTR group. The increase in BW observed during the trial in the TRT group significantly affected the BCS: from 60 days of administration, the TRT group showed significantly higher BCS (3.40–3.70; *p* < 0.0001) compared to the CTR group (Table 2).

The administration of the supplement significantly affected the arterial pressure. In particular, the CTR group showed a significant increase of diastolic pressure from T1 to T3 (*p* = 0.0037). The TRT group showed no significant variation in DBP values during the 90 days of observation. The systolic arterial pressure (SBP) was significantly reduced in the TRT group after 30 days of administration (*p* = 0.0106), but values were close to the physiological range. The dogs receiving the placebo showed a progressive increase of SBP values during the trial (*p* = 0.0026) (Table 3).

The cell blood count was not affected by the supplementation, and values were within normal ranges. No significant differences were observed during the trial in both groups.

No statistically significant differences were found in the creatinine level in both groups. In the CTR group, the BUN values increased during the trial from T1 onwards (*p* < 0.0001), while in the TRT group, the values were stable and decreased significantly at T3 (*p* < 0.0001). Moreover, in the TRT group there was a significant reduction (*p* = 0.0002) of phosphorus values and an increase in the CTR group (*p* = 0.0002) over time from T1 onwards. From T1 onwards, the supplement significantly increased serum protein and albumin concentrations. In fact, the TRT group showed an increase in concentrations (TP *p* = 0.0003; Alb *p* = 0.0001); in contrast, in the CTR group the values decreased (TP *p* = 0.0064; Alb *p* = 0.0002). There were no significant differences in A/G. The SDMA parameter showed significantly higher values in the CTR group at the end of the trial (*p* = 0.0454) (Table 4).

The supplement administration allows a reduction in proteinuria in the TRT group from T1 onwards (*p* = 0.0049), with a return to physiological values at T2. However, in the CTR group, the proteinuria (UPC) continued to worsen during the trial from T1 onwards (*p* = 0.0001). The urinary specify gravity (USG) showed a significant improvement from T1 onwards (*p* = 0.0001), returning to the normal range at T2 in the TRT group. Meanwhile, in the CTR group, the values continued to decrease (*p* = 0.0002) (Table 5).

CRP decreased in the TRT group from 60 days of administration and was significantly lower from T0 onwards (*p* = 0.0141). D-ROMS decreased in the TRT group from T1 onwards (*p* < 0.0001), while it increased at the same experimental time in the CTR group (*p* < 0.0001) (Table 6).

The iCa parameter showed no significant differences between groups (TRT 1.35–1.35; CTR 1.34–1.34 mmol/L) and remained in the physiological range during the trial (1.29–1.4 mmol/L).

## 4. Discussion

In the present study, we tested a supplement containing sodium bicarbonate, calcium carbonate, calcium lactate-gluconate, chitosan, *Lactobacillus acidophilus* D2/CSL, *Olea europaea* L. extract, and fructooligosaccharide (Renal Combi, Candioli srl, Beinasco, Turin, Italy). The product effectively slowed the progression of chronic kidney disease (CKD), as demonstrated by reductions in proteinuria, decreased urea levels, improved control of hyperphosphatemia and blood pressure, and lowered oxidative stress and inflammation. These findings align with a previous study in which the same supplement demonstrated significant improvements in renal function, phosphate levels, acid–base balance, and CKD-associated conditions such as inflammation and oxidative stress [16]. However, in the present study, we modified the dosing frequency from twice daily, as used previously, to once daily. This adjustment was made in consideration of the general clinical challenges faced by dogs with advanced CKD, who are often hyporexic and require multiple concurrent medications to manage uremia and hyperphosphatemia and support adequate nutritional intake [3,4,11,15]. Administering the supplement once daily offers a clear advantage for pet owners by simplifying the treatment regimen, which is especially important given the reduced appetite and low willingness to eat often seen in dogs with CKD, as well as the generally poor palatability of many supplements. Notably, using a single administration daily, we observed an improvement in urea levels in the TRT group compared to the CTR group, consistent with previous findings [16]. Other renal parameters, such as creatinine and SDMA, remained stable over time in the TRT group, while SDMA increased in the CTR group (Table 4). Even though creatinine and SDMA did not improve, probably because of the short observation time (90 days), this indicates a reduction of organ damage.

Additionally, we confirmed the effective control of other key markers relevant to the progression of chronic kidney disease (CKD), as outlined by IRIS guidelines. These include proteinuria (measured by the UPC ratio; Table 5), blood pressure (Table 3), and phosphorus levels (Table 4), although the trial duration may limit the assessment of long-term outcomes, such as disease progression, mortality, and quality of life.

Phosphatemia control is a key objective in the management of chronic kidney disease (CKD), often achieved through the use of phosphate binders, such as aluminum salts, calcium carbonate, calcium acetate, chitosan, and calcium lactate-gluconate, which reduce the intestinal absorption of phosphorus (P) and calcium (Ca) [2,3,11,12,13]. As previously reported [16,22], in our study the TRT group demonstrated better phosphorus control over time compared to the CTR group, without significant changes in ionized calcium (iCa) levels (Table 4).

This finding is particularly relevant, as effective phosphorus management was achieved with only once-daily supplementation. Hyperphosphatemia is frequently observed in dogs with advanced stages of CKD [1,2,3,4,23,24] and is commonly associated with disturbances in calcium-phosphorus (Ca–P) homeostasis [9,24]. Such imbalances negatively impact kidney function and overall survival [7,10,23]. Therefore, regular monitoring and control of phosphorus levels are critical components of CKD management [2,3,4,13,23].

Proteinuria and hypertension are critical factors to monitor and control in CKD patients, as emphasized by the IRIS guidelines [13]. Both are recognized as independent and significant contributors to disease progression in companion animals [1,2,3,4]. It is well established that a urinary protein-to-creatinine ratio (UPC) greater than one is associated with an increased risk of uremic crises, CKD progression, and mortality [25,26]. More recently, Miyakawa et al. [27] reported that even in non-azotemic proteinuric dogs, a UPC ratio greater than four was linked to a more rapid progression of kidney disease.

Hypertension is also an independent risk factor for CKD progression in dogs [3,4,28]. It has been associated with ocular, neurological, cardiac, and renal complications [29]. Importantly, it heightens the kidneys’ vulnerability to hypertensive damage [3,4,29,30].

The antioxidant component of the tested supplement, *Olea europaea*, may have contributed to the reduction of hypertension in the treated dogs. Olive leaf contains bioactive compounds such as oleuropein, oleracein, and oleanolic acid, and its antihypertensive and cholesterol-lowering properties are well established. Moreover, several studies have demonstrated that olive leaf extract can exert antihypertensive effects in patients with stage 1 hypertension [16,31,32]

Elevated blood pressure correlates with increased proteinuria, and since proteinuria itself contributes to ongoing renal damage, this association supports the therapeutic goal of blood pressure reduction to slow disease progression [29,33].

Alongside the observed control of proteinuria, dogs in the TRT group also showed improvements in total protein and serum albumin levels compared to the CTR group (Table 4). These findings may be attributed to a combination of dietary and feed supplement strategies, which have previously been shown to improve hypoalbuminemia and reduce proteinuria [22].

The improvement of patient conditions could be also linked to the presence of *Lactobacillus acidophilus* D2/CSL (CECT 4529) in the tested product, as also reported in a previous study [16]. In line with this, in one study carried out by Lippi and colleagues, dogs affected by CKD and treated with probiotics with *Lactobacillus* showed a significant improvement in glomerular filtration rate (GFR) and a significant reduction of UPC [34]. These studies highlight the beneficial role of probiotic supplementation in slowing the progression of CKD in dogs and emphasize the link between intestinal dysbiosis and kidney damage [35]. Research shows that gut microbiota contributes to the generation of several uremic toxins (i.e., indoxyl sulfate and p-cresol sulfate) that may promote renal disease [35,36]. Probiotic administration in dogs with CKD may enhance the elimination of uremic toxins via the intestines [34,36] and reduce systemic inflammation and proteinuria [37].

Improvement in serum albumin is closely related to the maintenance of BW and BCS, which are among the primary goals in the management of CKD patients [11,22,25]. In dogs with CKD and severe proteinuria, low BW and hypoalbuminemia are common and are associated with increased morbidity and mortality risk [26]. In our study, we observed increases in both BCS and BW over time in the TRT group (Table 2). In the later stages of CKD, muscle wasting is frequently seen due to uremia, which activates protein catabolic pathways [38], making it difficult for patients to maintain an ideal BCS [14,15,39]. Ensuring adequate food intake and stable BW as well as maintaining a BCS at or near 5/9 is therefore a key therapeutic objective [11].

Furthermore, adequate nutritional support and a higher BCS have been associated with longer survival in dogs with CKD [11,14,39,40]. The observed improvements in BCS and nutritional status in this study support the therapeutic efficacy of the tested supplement, even when administered once daily, and suggest it may positively influence appetite and overall patient condition.

Finally, consistent with previous findings [16], inflammatory and oxidative stress markers—C-reactive protein (CRP) and d-ROMs—significantly decreased over time in the TRT group, both in absolute terms and when compared to the CTR group during the trial (Table 5). Inflammation and oxidative stress are well-documented in dogs with CKD [41,42,43,44,45] and are closely linked to disease progression [46].

Oxidative stress contributes to renal tissue damage and chronic inflammation. Inflammatory cytokines, in turn, promote protein catabolism and suppress appetite, exacerbating cachexia and accelerating CKD progression [44]. Therefore, scientific evidence supports the inclusion of antioxidant-rich and omega-3 polyunsaturated fatty acid (ω-3 PUFA) supplementation in the dietary management of CKD patients to help reduce systemic inflammation and oxidative stress [11,44,47]. Indeed, Brown et al. (1998) [47] in short-term studies conducted on dogs with CKD observed that the administration of a ω-3 PUFA diet (particularly EPA and DHA and their precursor ALA), seems to decrease glomerular capillary pressure, with a renoprotective effect. Similarly, Vastolo et al. (2021) observed a significant reduction in creatinine levels in healthy dogs fed a ω-3 PUFA-rich diet, while no significant variations of creatinine levels were observed in dogs fed a ω-6 PUFA-rich diet [48,49].

Although our findings are consistent with previous reports, a formal comparative analysis was not the aim of the present study. The clinical outcomes observed here are consistent with those reported by Martello et al. (2021) [16], where the same supplement was administered twice daily but with the same total daily dosage. In the current study, the findings suggest that a once-daily regimen may be sufficient to achieve similar benefits in dogs with chronic kidney disease, potentially improving ease of use and owner compliance.

The study has some limitations. The limited sample size restricts the broader applicability of these findings, and future studies should include a larger cohort. Additionally, extending the duration of the study would be valuable to assess long-term outcomes, including prognosis and mortality related to uremic crises, particularly in dogs with advanced stages of CKD.

## 5. Conclusions

The tested supplement (Renal Combi, Candioli srl, Beinasco, Turin, Italy) in combination with a specific renal diet slowed CKD progression in dogs by reducing proteinuria, urea, hyperphosphatemia, blood pressure, oxidative stress, and inflammation. While these results are encouraging, they should be interpreted with caution, given the modest sample size and relatively short follow-up period. On the other hand, once-daily administration offers practical benefits, especially for dogs with advanced CKD who often have poor appetite and difficulty tolerating multiple daily medications. Simplifying the dosing regimen improves owner compliance, supporting consistent treatment. These findings endorse the supplement as a valuable adjunct in managing canine CKD, with the once-daily dose enhancing both patient tolerance and owner adherence. Further studies with larger cohorts and longer monitoring are warranted to confirm the clinical utility of this supplement as an adjunct in the management of canine CKD.

## Figures and Tables

**Table 1:** Composition of the feed supplement (Renal Combi, Candioli srl, Beinasco, Turin, Italy) and placebo used during the study.

Ingredients	%
**Feed supplement**	
Vitamin B12 1/1000	10
Vitamin E	0.002
Vitamin B6 Chlorhydrate	0.5
Vitamin C	5
Folic acid	0.2
Lactobacillus acidophilus D2/CSL	0.211
Olea europaea L.: Olive extract	2
Chitosan	8
Sodium Bicarbonate	6
Colloidal silica E551b	0.5
Calcium carbonate	26
Calcium lactate-gluconate	16
Fructo-oligosaccharides (Profeed^®^ Maxflow)	20
Optimizor Uranus	0.2
Maltodextrin	5.387
Total	100.000
**Placebo**	
Maltodextrin	95.00
Appetite stimulants	5.00
Total	100.000

**Table 2:** Mean values of body weight (BW; Kg) and body condition score (BCS: 5-point scale).

		TRT Group	CTR Group
Parameter	Time	Mean (Min–Max)	Mean (Min–Max)
**BW**	0	18.15 (7.1–29.8)	14.46 (6.2–27.2)
	1	18.21 (7.1–29.9)	14.44 (6.2–27.1)
	2	18.31 (7.2–30.0) **	14.43 (6.1–27.0)
	3	18.42 (7.5–30.1) **	14.42 (6.0–27.0)
**BCS**	0	3.20 (2–4)	2.73 (2–4)
	1	3.27 (2–4)	2.73 (2–4)
	2	3.40 (2–4) a	2.73 (2–4) b
	3	3.73 (3–5) A **	2.73 (2–4) B

** significant difference from T0 within group (*p* < 0.01); a, b—significant difference between groups at the same time (*p* < 0.05); A, B—significant difference between groups at the same time (*p* < 0.01).

**Table 3:** Mean value (mmHg) of diastolic blood pressure (DBP) and systolic blood pressure (SBP).

			TRT Group	CTR Group
Parameter	Time	Range	Mean (Min–Max)	Mean (Min–Max)
**DBP**	0	<95	84.67 (80–90) a	80.67 (80–90) b
	1		87.33 (80–90)	86.00 (80–90) **
	2		84.00 (80–90)	86.67 (80–90) **
	3		84.00 (80–90) A	90.00 (80–90) B **
**SBP**	0	<150	143.00 (130–150)	142.00 (140–150)
	1		136.00 (120–150) B *	150.00 (140–170) A **
	2		136.33 (130–150) B *	153.67 (140–180) A **
	3		134.00 (120–150) B **	156.67 (140–170) A **

* Significant difference from T0 within group (*p* < 0.05); ** significant difference from T0 within group (*p* < 0.01); a, b—significant difference between groups at the same time (*p* < 0.05); A, B—significant difference between groups at the same time (*p* < 0.01).

**Table 4:** Biochemical parameters: creatinine (CREA), blood urea nitrogen (BUN), phosphorus (P), total protein (TP), albumin (ALB), albumin/globulin (AG), and symmetric dimethylarginine (SDMA).

			TRT Group	CTR Group
Parameter	Time	Range	Mean (Min–Max)	Mean (Min–Max)
**CREA**	0	<1.4 mg/dL	3.16 (2.70–3.60)	3.24 (2.80–3.60)
	1		3.15 (2.60–3.80)	3.31 (2.50–3.90)
	2		3.12 (2.50–3.60)	3.27 (2.80–4.00)
	3		3.19 (2.70–3.80)	3.25 (2.60–3.90)
**BUN**	0	15–45 mg/dL	113.53 (82.80–150.80) b	131.46 (103.00–163.00) a
	1		111.22 (85.30–147.20) B	143.95 (119.00–182.60) A **
	2		110.93 (85.00–148.10) B	156.31 (124.10–189.70) A **
	3		106.67 (82.20–143.30) B **	168.93 (133.30–198.30) A **
**P**	0	3.5–4.7 mg/dL	7.64 (6.00–8.80)	8.11 (7.60–8.80)
	1		7.39 (5.60–8.70) B **	8.36 (7.60–9.10) A **
	2		7.18 (5.60–8.40) B **	8.60 (7.80–9.50) A **
	3		6.93 (5.30–8.30) B **	8.83 (7.90–9.80) A **
**PT**	0	6–7.5 mg/dL	5.59 (5.00–6.50)	5.70 (5.00–6.50)
	1		5.85 (5.10–6.80) **	5.50 (4.90–6.20) **
	2		6.01 (5.20–6.90) A **	5.33 (4.60–6.10) B **
	3		6.11 (5.20–6.90) A **	5.19(4.40–5.90) B **
**Alb**	0	2.5–4.2 mg/dL	2.01 (1.50–2.50)	1.97 (1.50–2.50)
	1		2.16 (1.60–2.70) a **	1.82 (1.20–2.50) b **
	2		2.28 (1.60–2.80) A **	1.70 (1.00–2.30) B **
	3		2.43 (1.70–2.90) A **	1.59 (0.70–2.30) B **
**A/G**	0	0.8–1.3 mg/dL	0.86 (0.70–1.00) b	1.01 (0.50–1.30) a
	1		0.85 (0.60–1.10) b	1.01 (0.60–1.30) a
	2		0.85 (0.70–1.10)	0.93 (0.60–1.30)
	3		0.86 (0.60–1.10)	0.87(0.50–1.30)
**SDMA**	0	<14 µg/dL	33.60 (29.00–38.00)	34.40 (30.00–39.00)
	1		33.53 (28.00–41.00)	35.60 (29.00–42.00)
	2		33.00 (27.00–38.00)	35.07 (30.00–43.00)
	3		32.40 (29.00–38.00) B	35.80 (29.00–42.00) A *

* Significant difference from T0 within group (*p* < 0.05); ** significant difference from T0 within group (*p* < 0.01); a, b—significant difference between groups at the same time (*p* < 0.05); A, B—significant difference between groups at the same time (*p* < 0.01).

**Table 5:** Urinary parameters: Urinary protein (PU), urinary protein-to-urinary creatinine (UPC), and urinary specify gravity (USG).

			TRT Group	CTR Group
Parameter	Time	Range	Mean (Min–Max)	Mean (Min–Max)
**PU**	0	0–150 mg/dL	239.92 (233.50–266.70)	235.73 (180.40–297.90)
	1		233.48 (194.20–264.80) **	243.59 (186.50–307.90) **
	2		228.54 (190.20–263.30) b **	255.85 (196.50–313.70) a **
	3		223.73 (185.80–255.90) B **	269.63 (205.00–332.50) A **
**UPC**	0	<0.5	0.66 (0.60–0.70)	0.73 (0.60–0.90)
	1		0.55 (0.40–0.70) B **	0.81 (0.60–1.10) A
	2		0.44 (0.30–0.70) B **	0.88 (0.60–1.20) A **
	3		0.35 (0.10–0.70) B **	0.96 (0.70–1.30) A **
**USG**	0	1020–1040	1017 (1012–1020) a	1014 (1011- 1018) b
	1		1019 (1012–1023) A **	1012 (1006–1018) B **
	2		1022 (1016–1025) A **	1010 (1004–1017) B **
	3		1024 (1021–1027) A **	1007 (999–1014) B **

** significant difference from T0 within group (*p* < 0.01); a, b—significant difference between groups at the same time (*p* < 0.05); A, B—significant difference between groups at the same time (*p* < 0.01).

**Table 6:** C-reactive protein (CRP) and reactive oxygen metabolite-derived compound (d-ROMs).

			TRT Group	CTR Group
Parameter	Time	Range	Mean (Min–Max)	Mean (Min–Max)
**CRP**	0	0–0.1 mg/dL	0.53 (0.18–1.03)	0.66 (0.04–1.06)
	1		0.50 (0.15–0.99)	0.67 (0.04–1.07)
	2		0.47 (0.10–0.94) b *	0.74 (0.04–1.21) a **
	3		0.44 (0.05–0.90) B **	0.84 (0.24–1.31) A **
**d-ROMs**	0	-	112.13 (100.60–125.30)	114.85 (102.80–129.60)
	1		105.53 (94.40–121.30) B **	120.45 (110.80–136.60) A **
	2		101.73 (89.90–118.30) B **	124.71 (115.30–136.60) A **
	3		96.19 (80.40–111.90) B **	129.38 (118.30–141.60) A **

* Significant difference from T0 within group (*p* < 0.05); ** significant difference from T0 within group (*p* < 0.01); a, b—significant difference between groups at the same time (*p* < 0.05); A, B—significant difference between groups at the same time (*p* < 0.01).

## Data Availability

Data are available on request to the corresponding author.

## Supplementary Materials

The following supporting information can be downloaded at: https://www.mdpi.com/article/10.3390/ani15192884/s1, Figure S1: Pathogenesis of Chronic Kidney Disease (CKD).
