# Supplementary figures and images for: Efficacy of a Once-Daily Supplement in Managing Canine Chronic Kidney Disease
Source: Animals (Basel). 2025 Oct 2;15(19):2884. doi: 10.3390/ani15192884 (PMC12524088; doi:10.3390/ani15192884)

## Supplementary file

Figure S1. Pathogenesis of Chronic Kidney Disease (CKD).

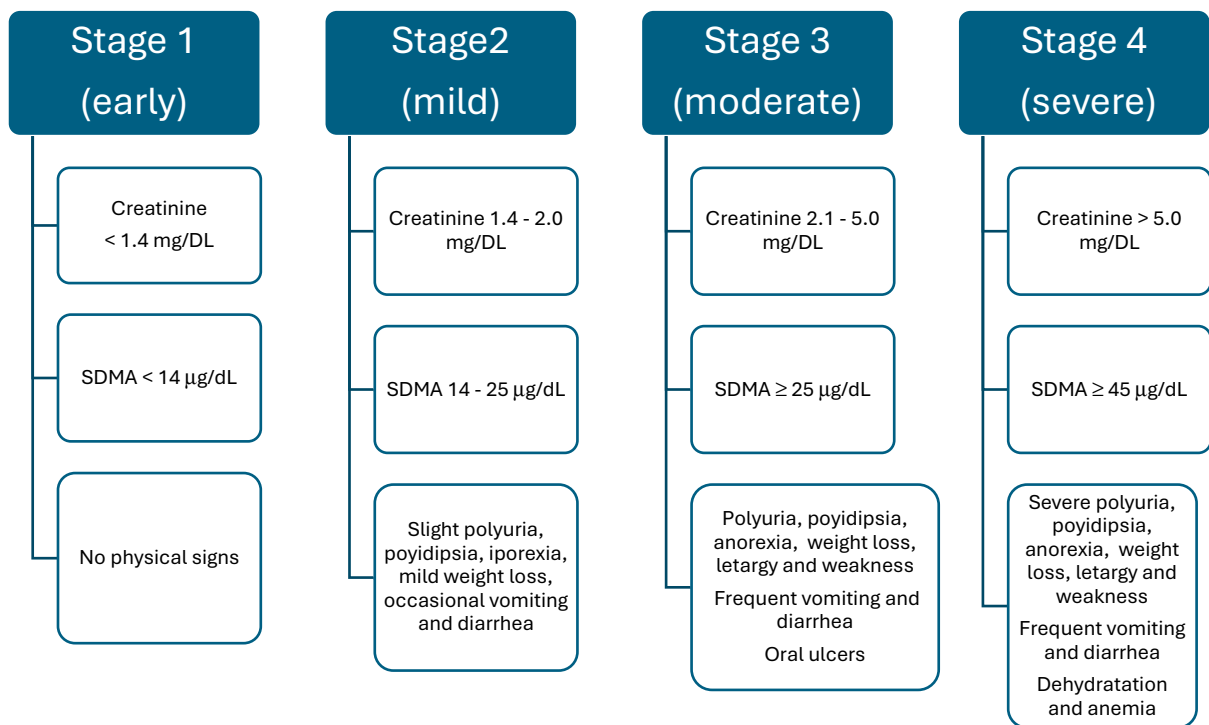

Supplement: Supplementary file 1 [file animals-15-02884-s001.zip › animals-3787785-supplementary.pdf]
